# A Case Report: Identification of a Pathogenic Microdeletion at Chromosome 21q21.3q22.13 Using Whole-Exome Sequencing and CNV Analysis in a Moroccan Child with Global Developmental Delay

**DOI:** 10.3390/genes16111280

**Published:** 2025-10-29

**Authors:** Farah Jouali, Ghyzlane El Haddoumi, Imane Antra, Rachid Benhida, Afaf Ben Itto, Jamal Fekkak

**Affiliations:** 1 ANOUAL CENTRE Laboratory, GENOMAF, Casablanca 20320, Morocco; fjouali@laboanoualcentre.ma (F.J.); iantra@laboanoualcentre.ma (I.A.); afaf.benitto@gmail.com (A.B.I.); jfekkak@hotmail.fr (J.F.); 2 Laboratory of Immunology and Biodiversity, Faculty of Sciences Ain Chock, Hassan II University, Casablanca 20100, Morocco; 3 Chemical and Biochemical Sciences, University Mohammed VI Polytechnic, Ben Guerir 43150, Morocco; rachid.benhida@um6p.ma; 4 Ibn Rochd, Appt No. 2, 4th Floor, Building 50, Morizgo District, Casablanca 20360, Morocco

**Keywords:** copy number variation, microdeletion, 21q21.3–q22.13, whole exome sequencing, genotype–phenotype correlation, SON

## Abstract

Copy number variations (CNVs) affecting the chromosomal region 21q21.3–q22.13 are rare and have been increasingly associated with neurodevelopmental abnormalities and multisystemic manifestations. In this study, we aimed to characterize the clinical, genomic, and genotype–phenotype correlations of a Moroccan child carrying a de novo microdeletion in this region. Whole exome sequencing (WES) was performed using sequencing-by-synthesis technology on the GenoLab M platform, and CNV detection was achieved through the SeqOne platform. Variant interpretation was conducted using the Integrative Genomics Viewer (IGV), and a custom gene–phenotype heatmap was generated in R (ComplexHeatmap and pheatmap) based on OMIM, ClinVar, and DECIPHER databases to prioritize candidate genes within the deleted segment. The patient presented with global developmental delay, microcephaly, psychomotor and staturo-ponderal retardation, facial dysmorphism, epilepsy responsive to treatment, and cerebral anomalies, including passive biventricular hydrocephalus and diffuse cortical atrophy. WES-CNV analysis identified a heterozygous de novo microdeletion of approximately 8.2 Mb in 21q21.3–q22.13, encompassing 124 clinically relevant genes. Integrated analysis confirmed the pathogenicity of the deletion and highlighted genotype–phenotype correlations, particularly implicating dosage-sensitive genes such as SON and RUNX1. This case underlines the clinical utility of combining WES, CNV analysis, and phenotype-based bioinformatic tools for diagnosing complex microdeletion syndromes, contributes to understanding genotype–phenotype relationships in 21q21.3–q22.13 deletions, and supports improved clinical interpretation and patient management.

## 1. Introduction

Microdeletions affecting the chromosome 21q21.3 to 21q22.13 are infrequent but carry notable clinical implications [1]. These deletions have been associated with complex neurodevelopmental phenotypes, most commonly global developmental delay (GDD). GDD is a heterogeneous clinical condition characterized by significant delays in multiple developmental domains, including motor, speech, cognition, and social functioning. It affects approximately 1–3% of children under the age of five and often reflects an underlying genetic etiology, particularly when associated with dysmorphic features, microcephaly, or neurological manifestations such as epilepsy [2].

In this study, we identified a copy number variant (CNV) consistent with the genetic diagnosis of 21q22.11q22.12 microdeletion syndrome (ORPHA: 261323). This CNV encompasses several genes, including RUNX1 and SON. Additionally, the CNV involves genes with LOC or open reading frame (ORF) designations, including LOC101928107, Chromosome 21 open reading frame 62 (C21orf62), and others.

Among the genes located within this critical region, SON emerges as a key contributor to the aforesaid phenotypes [3]. This gene (MIM #182465), located at 21q22.11, comprises 12 regular exons, among which exon 3 is the largest one, accounting for 82% of the coding region [4,5]. It encodes a DNA- and RNA-binding protein that functions as a splicing cofactor, playing a crucial role in RNA splicing, neurodevelopment, and cell cycle regulation [6]. The canonical isoform encoded by NM_138927.2 is a protein of 2426 amino acids that is highly conserved and ubiquitously expressed across all tissues and brain cells. This protein is a central component of nuclear speckles with SRRM2 (Serine/Arginine Repetitive Matrix 2) [7,8], and controls RNA splicing, gene transcription, and stem cell maintenance [9]. Deletion of SON results in double-strand DNA breakage, disrupted microtubule dynamics, neural abnormalities, and altered cell morphology [8,9,10]. Together, these effects support its pivotal role in preserving proper cell activity and genome integrity.

SON-related syndrome, also known as Zhu–Tokita–Takenouchi–Kim (ZTTK) syndrome (MIM #617140), is a multisystemic disorder that affects less than one in a million individuals worldwide [11]. Clinical manifestations are variable but commonly include developmental delay, intellectual disability, hypotonia, seizures, and brain malformations [11]. Additional features may encompass dysmorphic craniofacial traits, ocular problems, musculoskeletal anomalies, short stature, and congenital heart and genitourinary system defects [5,12]. Zhu et al. were the first to report this autosomal dominant hereditary disease by revealing de novo truncating mutations [13,14], and a dedicated website, ZTTK SON-Shine Foundation, is available at https://zttk.org/ (accessed on 2 August 2025).

Advancements in next-generation sequencing (NGS) technologies, especially whole-exome sequencing (WES), have significantly improved the detection of undiagnosed neurodevelopmental disorders. In particular, WES combined with CNV analysis allows the detection of both single-nucleotide variants (SNVs) and larger genomic rearrangements that may underlie complex phenotypes [15,16].

To the best of our knowledge, this is the first reported case of a Moroccan child carrying a novel 8.2 Mb microdeletion at chromosome 21q21.3–q22.13, encompassing 124 genes and consistent with a rare 21q microdeletion syndrome. Although deletions within chromosome 21q have been previously associated with neurodevelopmental phenotypes, the critical regions and their full clinical spectrum remain poorly defined. In this study, we combined whole-exome sequencing (WES) with copy-number-variation (CNV) calling to investigate the potential impact of this deletion on the patient’s clinical presentation and to refine the genotype–phenotype correlation. The integration of CNV analysis with phenotypic data and gene prioritization highlights the diagnostic value of WES-based CNV detection, even in the absence of detectable single-nucleotide variants. Moreover, this work provides new insights into the contribution of dosage-sensitive genes, particularly SON and RUNX1, to the observed phenotype, thereby expanding the molecular and clinical understanding of 21q21.3–q22.13 deletions.

## 2. Materials and Methods

### 2.1. Clinical Features of the Patient

The patient, a two-year-old Moroccan child born to a non-consanguineous marriage, is the youngest of four siblings from the same parents. Clinically, the patient displayed global psychomotor and stature-ponderal retardation, alongside microcephaly, facial dysmorphia, and pharmaco-sensitive epilepsy. Imaging via brain MRI (magnetic resonance imaging) identified passive biventricular hydrocephalus and diffuse cortical atrophy.

### 2.2. Genomic Analysis

#### 2.2.1. DNA Extraction

Genomic DNA was extracted from peripheral blood using a solid-phase extraction (SPE) method (Wizard^®^ Genomic DNA Purification Kit, Promega Corporation, Madison, WI, USA), following the manufacturer’s protocol.

#### 2.2.2. Whole Exome Sequencing (WES) and Bioinformatic Analysis

The libraries were prepared on the Magnis NGS prep system using the SureSelect XT HS2 DNA Target Enrichment V8 kit with a version B0 protocol, May 2023 (Agilent Technologies, Santa Clara, CA, USA), and then quantified using the Qubit HS (Thermo Fisher Scientific, Waltham, MA, USA) and the Agilent D2400 bio-Analyzer.

Whole exome sequencing was performed using the Genolab M platform, generating 150 bp paired-end reads (2 × 150 bp), in accordance with the manufacturer’s protocol (Genemind Biosciences Co., Shenzhen, China). The raw sequencing data (FASTQ files) were uploaded to the SeqOne platform to provide a whole bioinformatic analysis, including read alignment to the human reference genome (GRCh37/hg19), variant calling, and annotation (gene, functional effect, and clinical impact). Candidate variants were then classified according to the American College of Medical Genetics and Genomics (ACMG) criteria.

#### 2.2.3. CNV Detection and Visualization

CNV analysis was performed to detect and identify potential structural variants, thereby annotating large copy number alterations. Manual confirmation and visualization of this deletion were performed using Integrative Genomics Viewer (IGV).

#### 2.2.4. SNP-CGH Array

A SNP-CGH array was performed to confirm the presence of this CNV at chromosome cytobands 21q21.3–q22.13. The Infinium Global Diversity Array with Cytogenetics kit (Illumina Inc., San Diego, CA, USA) was used. Genomic DNA was amplified, fragmented, and hybridized onto the array according to the manufacturer’s instructions. Data analysis was carried out using the NxClinical software 6.0 (BioDiscovery, Panchkula, India).

#### 2.2.5. Gene–Phenotype Prioritization and Heatmap Generation

Genes encompassed within the deleted region were extracted based on genomic coordinates. Their known clinical associations were curated through manual review of OMIM, DECIPHER, ClinVar, and relevant publications indexed in PubMed. Each gene was then scored across five phenotypic categories: Neurodevelopmental disorders, Immunodeficiency, Cardiac anomalies, Epilepsy, and Parkinsonism using a binary matrix (1 = known association, 0 = no established link). The outputs were visualized as a heatmap using the ComplexHeatmap and pheatmap packages in R (v4.3.0).

### 2.3. Workflow Overview

**Figure 1:**
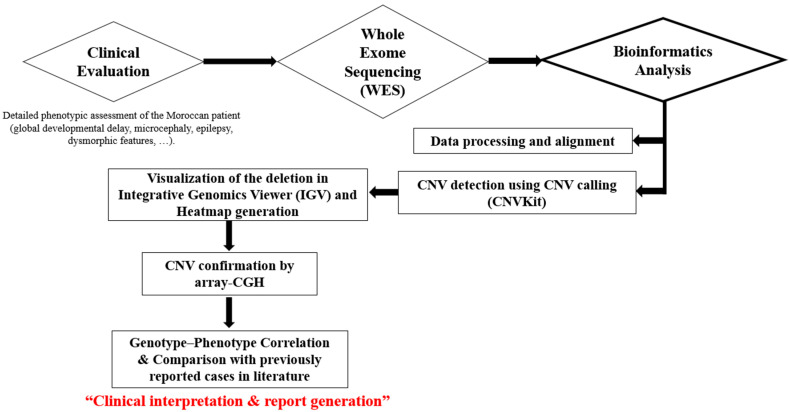
Overview of the analytical workflow used in this study. Legend: The pipeline begins with the clinical evaluation of the Moroccan patient, including a detailed assessment of neurodevelopmental and dysmorphic features. Whole-exome sequencing (WES) was then performed using the GenoLab M platform. Sequencing data were processed, aligned, and analyzed for copy number variations (CNVs) using CNVkit. The detected CNV was visualized in Integrative Genomics Viewer (IGV) and confirmed by comparative genomic hybridization (CGH). A phenotype–gene heatmap was generated to explore the genotype–phenotype correlation within the deleted region (21q21.3–q22.13).

To provide a clear overview of the analytical strategy, we summarized the methodological pipeline in Figure 1. The workflow includes the main steps from clinical assessment to genomic data analysis, CNV confirmation, and genotype–phenotype correlation.

## 3. Results

**Figure 2:**
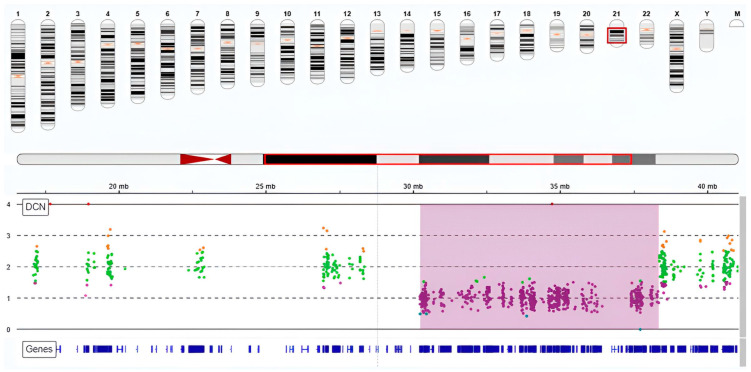
Copy number profile of chromosome 21 showing a de novo microdeletion. Legend: This figure illustrates the copy number variation (CNV) profile for chromosome 21 in the proband, showing a de novo heterozygous microdeletion spanning 21q21.3–q22.13. The purple shaded area highlights the deleted region, extending from approximately 30.3 Mb to 38.5 Mb. The y-axis represents log2 ratio values obtained from CNV calling using ExomeDepth. Values below 1 indicate loss of copy number. Below, gene tracks mark annotated genes within the deleted interval, including GRIK1, TIAM1, and ERG. The deletion is visually validated by the reduction in sequencing read depth across the affected region as displayed in the Integrative Genomics Viewer (IGV). This integrated analysis confirms the pathogenicity of the large CNV identified.

Whole-exome sequencing (WES) analysis did not identify any pathogenic single-nucleotide variants (SNVs) that could explain the patient’s phenotype. However, CNV calling based on WES read-depth data revealed a heterozygous microdeletion on the long arm of chromosome 21, specifically spanning the 21q21.3–q22.13 region (Figure 2). This deletion measures approximately 8.2 Mb, extending from position 30.3 Mb to 38.5 Mb (Table 1), and affects several clinically relevant genes, including SON and RUNX1, and has a sample frequency of 0.81%. CNV calling thresholds and quality metrics were applied using SeqOne, and visualization and confirmation were performed with IGV (Table 2).

### Phenotype–Genotype Correlation

**Figure 3:**
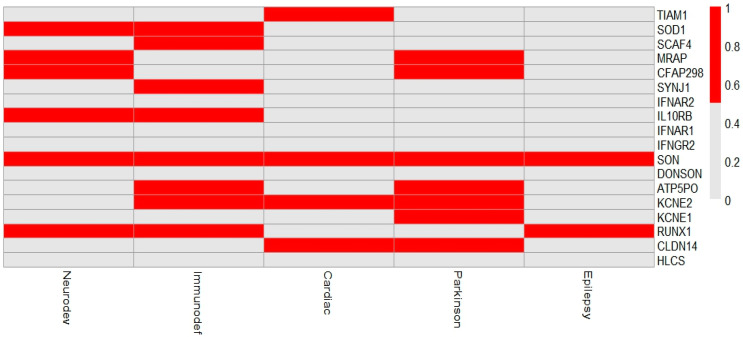
Heatmap of gene–phenotype associations in the 21q21.3–q22.13 deletion. Legend: This figure presents a heatmap summarizing the known phenotype associations of genes located within the deleted 21q21.3–q22.13 region. Rows correspond to individual genes, while columns represent clinical phenotypes, including neurodevelopmental disorders, immunodeficiency, cardiac anomalies, Parkinson’s disease, and epilepsy. Red cells indicate the presence of a documented gene–phenotype association, while gray cells denote the absence of such evidence. Genes such as SON and RUNX1 are linked to multiple phenotypic categories, suggesting their potential contribution to the complex clinical presentation of the patient. The visualization highlights the pleiotropic impact of the deleted genes and underscores the importance of considering multi-systemic involvement in patients carrying large pathogenic deletions.

Figure 3 illustrates the distribution of phenotype associations for each gene in the deleted region.

To contextualize this result, we compared the present 8.2 Mb 21q21.3–q22.13 deletion (arr[GRCh37]21q21.3q22.13(30093156_38340656)x1) with previously reported 21q deletions and SON-related cases. Overlapping large interstitial deletions involving 21q22 that include dosage-sensitive genes such as RUNX1 have been associated with neurodevelopmental delay, dysmorphism, and hematologic abnormalities, and contiguous-gene 21q22 deletions of comparable size (~7–8 Mb) have been reported previously. Conversely, most SON-related reports describe single-gene loss-of-function (frameshift/nonsense) rather than large deletions; these studies nevertheless support the link between SON haploinsufficiency and neurodevelopmental features (developmental delay, intellectual disability, hypotonia, seizures). We therefore present a comparative table (Table 3) summarizing our case and representative previously published cases to clarify overlapping features and differences

## 4. Discussion

Over the past decade, the study of structural variation in the human genome has emerged as one of the fastest-growing fields in genetics. Among these variations, copy number variants (CNVs) occur more frequently than previously anticipated. While CNV contributes to interindividual phenotypic variability, they are increasingly recognized for their role in modulating the expressivity and severity of chromosomal disorders. Consequently, understanding the implications of CNV in human disease requires a careful assessment of its potential phenotypic effects. Notably, CNVs involving deletions or duplications of dosage-sensitive genes, or their regulatory regions, can disrupt gene expression or function, often resulting in significant clinical consequences [22,23].

A compelling example of the clinical relevance of CNV centers on the 21q22.11q22.12 microdeletion, a rare genetic abnormality with a distinct phenotypic profile. This syndrome results from a partial deletion of the long arm of chromosome 21 and is characterized by prenatal and postnatal growth retardation, short stature, intellectual disability, developmental delay with severe language impairment, thrombocytopenia, and craniofacial dysmorphism that may include microcephaly, downward slanting palpebral fissures, broad or depressed nasal bridge, and epicanthal folds [24]. Brain abnormalities on MRI (such as agenesis of the corpus callosum), behavioral disturbances, and seizures may be associated [24,25]. Katzaki et al. reported three new patients with overlapping de novo interstitial deletions involving cytoband 21q22 and including the RUNX1 gene, presenting with severe developmental delay, dysmorphic features, behavioral problems, and thrombocytopenia [26,27,28,29].

According to the current literature and genetic databases, the CNV identified in this case has not been previously reported and is classified as pathogenic according to ACMG guidelines. This deletion includes both the RUNX1 and SON genes, which are functionally interacting and contribute to the patient’s combined neurodevelopmental and hematologic phenotype. Both SON and RUNX1 have established haploinsufficiency evidence according to the ClinGen dosage sensitivity map. Haploinsufficiency of RUNX1 leads to reduced transcriptional regulation of key genes involved in hematopoiesis, resulting in hereditary thrombocytopenia, characterized by a decreased number of circulating platelets. Furthermore, impaired RUNX1 function disrupts the normal differentiation and proliferation of hematopoietic stem and progenitor cells, thereby increasing the risk of developing hematologic malignancies, particularly myeloid leukemias [30,31,32], while SON is implicated in Zhu–Tokita–Takenouchi–Kim syndrome [11], which is a rare neurodevelopmental disorder characterized by a heterogeneous spectrum of phenotypic and clinical features [4,6]. Additionally, SON emerged as a key node across multiple phenotypic domains, as shown in the generated heatmap, highlighting its central role in neurodevelopmental, epileptic, and immune-related pathways, which supports its contribution to the patient’s clinical presentation (Figure 3).

The presence of both SON and RUNX1 within the deleted segment provides a plausible genetic basis for the proband’s combined neurodevelopmental and hematologic features. Previous studies of 21q deletions, including RUNX1, describe thrombocytopenia and predisposition to myeloid malignancies, while separate reports of SON loss-of-function variants reproduce the core ZTTK neurodevelopmental phenotype, together supporting the pathogenicity of the present contiguous deletion [18]. A direct comparison of our case with previously published key cases is provided in Table 3, highlighting similarities and differences in clinical features, genetic findings, and outcomes. This comparison facilitates the contextualization of our findings within the existing literature and underscores the unique aspects of the present case.

Overall, the integration of molecular findings with clinical observations is crucial for refining the diagnosis of rare disorders like ZTTK and microdeletion syndromes. Although these conditions share overlapping features, such as global developmental delay, intellectual disability, hypotonia, and dysmorphic facial features, they are genetically distinct. While mutations in the SON gene have been recognized as causative of ZTTK [14], the 21q21.3–q22.13 microdeletion syndrome arises from a larger chromosomal deletion implying multiple genes leading to a more complex and heterogeneous picture [1].

In this report, our patient fits the known clinical presentation of microdeletion syndrome and highlights the broader gene loss seen in 21q microdeletions. To the best of our knowledge, this is the first case to be reported in Morocco, broadening the geographical and ethnic spectrum. The collected data emphasize the importance of establishing genotype-phenotype correlations and shed light on the critical role of genetic testing in diagnosing rare and atypical neurodevelopmental disorders across diverse populations. These findings support the pathogenicity of the identified CNV and its contribution to the neurodevelopmental and hematologic abnormalities observed.

## 5. Conclusions

Numerous regions of the human genome, comprising hundreds of genes, exhibit copy-number variations through recurrent deletions or duplications, highlighting their clinical relevance in shaping phenotypic variability in aneuploidy and other chromosomal disorders. In this case report, we identified a de novo heterozygous CNV in the 21q21.3–q22.13 region, encompassing key genes including SON and RUNX1, which are functionally relevant to neurodevelopmental and hematopoietic pathways. The proband’s clinical presentation illustrates the complexity and heterogeneity of microdeletion syndromes, reinforcing the syndromic nature of this CNV. Integration of whole-exome sequencing with CNV analysis, functional annotation, and gene–phenotype heatmaps further supports the pathogenicity of the deletion and its contribution to the combined neurodevelopmental and hematologic phenotype. Given that the clinical features can vary depending on the size of the deletion and the specific genes involved, close clinical follow-up is essential for monitoring potential comorbidities. These findings contribute to the growing literature on gene haploinsufficiency as a driver of multisystemic phenotypes and emphasize the need for ongoing variant curation to refine our understanding of genotype-phenotype correlations. Future functional studies will be crucial to support targeted therapies and personalized care strategies. In this context, genetic counseling remains integral, guiding reproductive choices and long-term clinical management, particularly in regions with limited access to advanced genomic technologies.

## Figures and Tables

**Table 1:** Data about the identified CNV.

Genomic Coordinates	CNV Size (kb)	Number of Genes	Classification	Disorder
arr[GRCh37] 21q21.3q22.13 (30093156_38340656)x1	8248	124	Pathogenic	21q22.11q22.12 Microdeletion syndrome

This table summarizes the genomic characteristics of the copy number variant (CNV) detected in the patient. Genomic coordinates are reported according to GRCh37. The CNV encompasses a region from 21q21.3 to q22.13, with an approximate size of 8248 kb, spanning 124 annotated genes. Based on current clinical and molecular evidence, this CNV is classified as pathogenic. The disorder associated with this variant is a 21q22.11q22.12 microdeletion syndrome, a rare genomic condition characterized by variable clinical features.

**Table 2:** Overview of the 124 genes within the 21q21.3–q22.13 microdeletion.

List of Genes Spanning the 21q21.3–q22.13 Deleted Region
N6AMT1, LTN1, RWDD2B, USP16, CCT8, MAP3K7CL, LINC00189, BACH1, BACH1-IT2, BACH1- IT3, GRIK1-AS2, GRIK1, GRIK1-AS1, CLDN17, LINC00307, CLDN8, KRTAP24-1, KRTAP25-1, KRTAP26-1, KRTAP27-1, KRTAP23-1, KRTAP13-2, MIR4327, KRTAP13-1, KRTAP13-3, KRTAP13-4, KRTAP15-1, KRTAP19-1, KRTAP19-2, KRTAP19-3, KRTAP19-4, KRTAP19-5, KRTAP19-6, KRTAP19- 7, KRTAP22-2, KRTAP6-3, KRTAP6-2, KRTAP22-1, KRTAP6-1, KRTAP20-1, KRTAP20-4, KRTAP20-2, KRTAP20-3, KRTAP21-3, KRTAP21-2, KRTAP21-1, KRTAP8-1, KRTAP7-1, KRTAP11-1, KRTAP19-8, TIAM1, LOC150051, SOD1, SCAF4, HUNK, LINC00159, MIS18A, MIS18A-AS1, MRAP, URB1, SNORA80A, URB1-AS1, EVA1C, TCP10L, CFAP298-TCP10L, CFAP298, SYNJ1, PAXBP1-AS1, PAXBP1, C21orf62-AS1, C21orf62, LINC01690, OLIG2, LINC00945, OLIG1,LOC101928107, LINC01548, IFNAR2, IL10RB-DT, IL10RB, IFNAR1,IFNGR2, TMEM50B, DNAJC28, GART, MIR6501, SON, DONSON, CRYZL1, ITSN1, ATP5PO, LINC00649, LOC101928126, SLC5A3, MRPS6, LINC00310, KCNE2, SMIM11A, FAM243A, SMIM34A, KCNE1, RCAN1, CLIC6, LINC00160, LINC01426, RUNX1, RUNX1-IT1, LOC100506403, MIR802, PPP1R2P2, LOC101928269, LINC01436, SETD4, CBR1, LOC100133286, LOC105369306, CBR3, CBR3-AS1, DOP1B, MORC3, CHAF1B, CLDN14, SIM2, HLCS.

This table lists the 124 protein-coding genes and non-coding elements located within the deleted region identified on chromosome 21q21.3–q22.13. The gene set includes structural, regulatory, and signaling elements, several of which have been previously implicated in neurodevelopmental processes, immune regulation, and congenital anomalies. The heterogeneity of gene functions may explain the complex clinical phenotype observed in affected individuals. The list combines both well-characterized genes and poorly annotated loci, underlining the potential contribution of multiple genomic factors to disease manifestation.

**Table 3:** Comparison of the present case with representative previously reported 21q deletions and SON-associated cases.

Study (Year)— Reference	Genomic Coordinates/CNV Size (hg19)	Key Genes Included (RUNX1/SON)	Main Clinical Features Reported	Detection Method
Our study (2025)	arr[GRCh37] 21q21.3–q22.13 (30093156–38340656)—~8.2 Mb	124 genes; includes SON and RUNX1	Global developmental delay, microcephaly, facial dysmorphism, epilepsy, cortical atrophy, growth delay	WES with CNV calling: confirmed by SNP-CGH array
Braddock–Carey et al., 2016 (AJMG A) [17]	chr21:31391467–39118687 (hg19)—~7.7 Mb	Includes RUNX1 and multiple neighboring genes	Severe developmental delay, dysmorphism, thrombocytopenia, behavioral problems	aCGH/microarray
Shinawi et al., 2008 (Blood) [18]	reported constitutional deletions involving 21q22 (various sizes)	RUNX1 included in several patients	Hereditary thrombocytopenia, predisposition to myeloid malignancies; some neuro features	aCGH/cytogenetics
Li et al., 2018 (BMC Gnomiques) [19]	various microdeletions on chr21 (reviewed cases)	variable (some include the SON region)	Developmental delay, microcephaly, seizures, congenital anomalies	aCGH/CNV studies
Tang et al., 2023 (Molecular Genetics and Genomic Medicine) [20]	Single-gene SON loss-of-function (frameshift/nonsense), not large deletion	SON (variants e.g., c.1845_1870del26)	Developmental delay, seizures, brain anomalies	Exome/targeted sequencing
Pietrobattista et al., 2023 (Genes) [21]	(review)	SON (discussed)	Broad ZTTK phenotype spectrum, hypotonia, seizures, multisystemic features	Review/database analysis

## Data Availability

The datasets generated and analyzed during the current study are available from the corresponding author on reasonable request.

## Abbreviations

The following abbreviations are used in this manuscript:

CNV	Copy Number Variant
WES	Whole Exome Sequencing
GDD	Global Developmental Delay
SNP-CGH	Single Nucleotide Polymorphism—Comparative Genomic Hybridization
MRI	Magnetic Resonance Imaging
IGV	Integrative Genomics Viewer
ACMG	American College of Medical Genetics and Genomics
ORF	Open Reading Frame
LOC	Locus
ZTTK	Zhu–Tokita–Takenouchi–Kim
NGS	Next-Generation Sequencing
SNV	Single-Nucleotide Variant

## References

[1] Carvill G.L., Mefford H.C. (2013). Microdeletion Syndromes. Curr. Opin. Genet. Dev..

[2] Modha B. (2021). Global Developmental Delay and Its Considerations in Paediatric Dental Care—A Case Report. Oral.

[3] Tokita M.J., Braxton A.A., Shao Y., Lewis A.M., Vincent M., Küry S., Besnard T., Isidor B., Latypova X., Bézieau S. (2016). De Novo Truncating Variants in SON Cause Intellectual Disability, Congenital Malformations, and Failure to Thrive. Am. J. Hum. Genet..

[4] Dingemans A.J.M., Truijen K.M.G., Kim J.-H., Alaçam Z., Faivre L., Collins K.M., Gerkes E.H., van Haelst M., van de Laar I.M.B.H., Lindstrom K. (2022). Establishing the Phenotypic Spectrum of ZTTK Syndrome by Analysis of 52 Individuals with Variants in SON. Eur. J. Hum. Genet..

[5] Kim J.-H., Shinde D.N., Reijnders M.R.F., Hauser N.S., Belmonte R.L., Wilson G.R., Bosch D.G.M., Bubulya P.A., Shashi V., Petrovski S. (2016). De Novo Mutations in SON Disrupt RNA Splicing of Genes Essential for Brain Development and Metabolism, Causing an Intellectual-Disability Syndrome. Am. J. Hum. Genet..

[6] Slezak R., Smigiel R., Rydzanicz M., Pollak A., Kosinska J., Stawinski P., Malgorzata Sasiadek M., Ploski R. (2020). Phenotypic Expansion in Zhu-Tokita-Takenouchi-Kim Syndrome Caused by de Novo Variants in the SON Gene. Mol. Genet. Genomic. Med..

[7] Ilik İ.A., Malszycki M., Lübke A.K., Schade C., Meierhofer D., Aktaş T. (2020). SON and SRRM2 Are Essential for Nuclear Speckle Formation. eLife.

[8] Xu S., Lai S.-K., Sim D.Y., Ang W.S.L., Li H.Y., Roca X. (2022). SRRM2 Organizes Splicing Condensates to Regulate Alternative Splicing. Nucleic Acids Res..

[9] Ueda M., Matsuki T., Fukada M., Eda S., Toya A., Iio A., Tabata H., Nakayama A. (2020). Knockdown of Son, a Mouse Homologue of the ZTTK Syndrome Gene, Causes Neuronal Migration Defects and Dendritic Spine Abnormalities. Mol. Brain.

[10] Ahn E.-Y., DeKelver R.C., Lo M.-C., Nguyen T.A., Matsuura S., Boyapati A., Pandit S., Fu X.-D., Zhang D.-E. (2011). SON Controls Cell Cycle Progression by Coordinated Regulation of RNA Splicing. Mol. Cell.

[11] Kushary S.T., Revah-Politi A., Barua S., Ganapathi M., Accogli A., Aggarwal V., Brunetti-Pierri N., Cappuccio G., Capra V., Fagerberg C.R. (2021). ZTTK Syndrome: Clinical and Molecular Findings of 15 Cases and a Review of the Literature. Am. J. Med. Genet. Part A.

[12] Yang L., Yang F. (2020). A de Novo Heterozygous Variant in the SON Gene Is Associated with Zhu-Tokita-Takenouchi-Kim Syndrome. Mol. Genet. Genom. Med..

[13] Zhu X., Petrovski S., Xie P., Ruzzo E.K., Lu Y.-F., McSweeney K.M., Ben-Zeev B., Nissenkorn A., Anikster Y., Oz-Levi D. (2015). Whole-Exome Sequencing in Undiagnosed Genetic Diseases: Interpreting 119 Trios. Genet. Med..

[14] Yang Y., Xu L., Yu Z., Huang H., Yang L. (2019). Clinical and Genetic Analysis of ZTTK Syndrome Caused by SON Heterozygous Mutation c.394C>T. Mol. Genet. Genom. Med..

[15] Hamanaka K., Miyake N., Mizuguchi T., Miyatake S., Uchiyama Y., Tsuchida N., Sekiguchi F., Mitsuhashi S., Tsurusaki Y., Nakashima M. (2022). Large-Scale Discovery of Novel Neurodevelopmental Disorder-Related Genes through a Unified Analysis of Single-Nucleotide and Copy Number Variants. Genome Med..

[16] Tilemis F.-N., Marinakis N.M., Veltra D., Svingou M., Kekou K., Mitrakos A., Tzetis M., Kosma K., Makrythanasis P., Traeger-Synodinos J. (2023). Germline CNV Detection through Whole-Exome Sequencing (WES) Data Analysis Enhances Resolution of Rare Genetic Diseases. Genes.

[17] Braddock S.R., South S.T., Schiffman J.D., Longhurst M., Rowe L.R., Carey J.C. (2016). Braddock-Carey Syndrome: A 21q22 Contiguous Gene Syndrome Encompassing RUNX1. Am. J. Med. Genet. A.

[18] Shinawi M., Erez A., Shardy D.L., Lee B., Naeem R., Weissenberger G., Chinault A.C., Cheung S.W., Plon S.E. (2008). Syndromic Thrombocytopenia and Predisposition to Acute Myelogenous Leukemia Caused by Constitutional Microdeletions on Chromosome 21q. Blood.

[19] Li W., Wang X., Li S. (2018). Investigation of Copy Number Variations on Chromosome 21 Detected by Comparative Genomic Hybridization (CGH) Microarray in Patients with Congenital Anomalies. Mol. Cytogenet..

[20] Tang S., You J., Liu L., Ouyang H., Jiang N., Duan J., Li C., Luo Y., Zhang W., Zhan M. (2023). Expanding the Mutational Spectrum of ZTTK Syndrome: A de Novo Variant with Global Developmental Delay and Malnutrition in a Chinese Patient. Mol. Genet. Genom. Med..

[21] The Expanding Phenotype of ZTTK Syndrome Due to the Heterozygous Variant of SON Gene Focusing on Liver Involvement: Patient Report and Literature Review. https://www.mdpi.com/2073-4425/14/3/739.

[22] de Smith A.J., Trewick A.L., Blakemore A.I.F. (2010). Implications of Copy Number Variation in People with Chromosomal Abnormalities: Potential for Greater Variation in Copy Number State May Contribute to Variability of Phenotype. HUGO J..

[23] Stranger B.E., Forrest M.S., Dunning M., Ingle C.E., Beazley C., Thorne N., Redon R., Bird C.P., de Grassi A., Lee C. (2007). Relative Impact of Nucleotide and Copy Number Variation on Gene Expression Phenotypes. Science.

[24] Briegel W., Hoyer J. (2020). Psychiatric Disorders and Distal 21q Deletion—A Case Report. Int. J. Environ. Res. Public Health.

[25] Errichiello E., Novara F., Cremante A., Verri A., Galli J., Fazzi E., Bellotti D., Losa L., Cisternino M., Zuffardi O. (2016). Dissection of Partial 21q Monosomy in Different Phenotypes: Clinical and Molecular Characterization of Five Cases and Review of the Literature. Mol. Cytogenet..

[26] Katzaki E., Morin G., Pollazzon M., Papa F.T., Buoni S., Hayek J., Andrieux J., Lecerf L., Popovici C., Receveur A. (2010). Syndromic Mental Retardation with Thrombocytopenia Due to 21q22.11q22.12 Deletion: Report of Three Patients. Am. J. Med. Genet A.

[27] van der Crabben S., van Binsbergen E., Ausems M., Poot M., Bierings M., Buijs A. (2010). Constitutional *RUNX1* Deletion Presenting as Non-Syndromic Thrombocytopenia with Myelodysplasia: 21q22 *ITSN1* as a Candidate Gene in Mental Retardation. Leuk. Res..

[28] Christensen R.D., Wiedmeier S.E., Yaish H.M. (2013). A Neonate with Congenital Amegakaryocytic Thrombocytopenia Associated with a Chromosomal Microdeletion at 21q22.11 Including the Gene RUNX1. J. Perinatol..

[29] Béri-Dexheimer M., Latger-Cannard V., Philippe C., Bonnet C., Chambon P., Roth V., Grégoire M.-J., Bordigoni P., Lecompte T., Leheup B. (2008). Clinical Phenotype of Germline RUNX1 Haploinsufficiency: From Point Mutations to Large Genomic Deletions. Eur. J. Hum. Genet..

[30] Vukadin L., Kim J.-H., Park E.Y., Stone J.K., Ungerleider N., Baddoo M.C., Kong H.K., Richard A., Tran J., Giannini H. (2021). SON Inhibits Megakaryocytic Differentiation via Repressing RUNX1 and the Megakaryocytic Gene Expression Program in Acute Megakaryoblastic Leukemia. Cancer Gene Ther..

[31] Vukadin L., Park E.y., Kim J.H., Ahn E.E.Y. (2018). Abstract 1482: SON Represses RUNX1 Expression and Impairs Megakaryocytic Differentiation in Down Syndrome Acute Megakaryoblastic Leukemia (DS-AMKL). Cancer Res..

[32] North T.E., de Bruijn M.F.T.R., Stacy T., Talebian L., Lind E., Robin C., Binder M., Dzierzak E., Speck N.A. (2002). Runx1 Expression Marks Long-Term Repopulating Hematopoietic Stem Cells in the Midgestation Mouse Embryo. Immunity.

